# Genomic Variability of Canine Parvoviruses from a Selected Population of Dogs and Cats in Sri Lanka

**DOI:** 10.3390/pathogens10091102

**Published:** 2021-08-29

**Authors:** Rasika Jinadasa, Sayani Ghosh, Simon Hills, Thushini Premalal, Ushani Atapattu, Manohari Fuward, Wasantha Kalupahana, Magdalena Dunowska

**Affiliations:** 1 Department of Veterinary Pathobiology, Faculty of Veterinary Medicine & Animal Science, University of Peradeniya, Peradeniya 20400, Sri Lanka; thushiniwanthila@gmail.com (T.P.); ushani40@yahoo.com (U.A.); anilwkalupahana@yahoo.com (W.K.); 2 School of Veterinary Science, Massey University, Palmerston North 4410, New Zealand; S.Ghosh1@massey.ac.nz; 3 Institute of Agriculture and Environment, Massey University, Palmerston North 4410, New Zealand; S.F.Hills@massey.ac.nz; 4 Government Veterinary Hospital, Gatambe, Peradeniya 20400, Sri Lanka; gvh.gatambe@gmail.com

**Keywords:** canine parvovirus, CPV-2, CPV-2 subtypes, hyplotype network, CPV-2 host, CPV-2 evolution, viral evolution, Sri Lanka

## Abstract

The aim of the study was to identify canine parvovirus type 2 (CPV-2) subtypes circulating among a selected population of domestic dogs and cats in Sri Lanka and to investigate the evolutionary patterns among Sri Lankan viruses in the context of contemporary global CPV-2 sequences. Altogether, 40/61 (65.6%) samples tested were positive for CPV-2 DNA, including 31/48 (64.6%) dogs and 9/13 (69%) cats. All three subtypes (CPV-2a, CPV-2b and CPV-2c) were detected, with CPV-2a being most common. International median joining haplotype network of 291 CPV-2 sequences suggested that there was little barrier for CPV-2 moving between different geographical regions worldwide, including Sri Lanka, and that there was no correlation between the genetic structure within the molecular network and the decade of sample collection. By contrast, there was correlation between CPV-2 subtype and genetic structure, both within the international network and within the network built from 31 Sri Lankan CPV-2 sequences only. The structure within the latter was not correlated with the location of the veterinary clinic where the samples were submitted, the age or species of the host. Altogether, we have shown that there is considerable variability of CPV-2 genotypes circulating in Sri Lanka, which is likely driven by both local evolution and introduction from other countries. The similarity of CPV-2 obtained from cats and dogs suggests that cats may play a role in the epidemiology of CPV-2 in Sri Lanka.

## 1. Introduction

Canine parvovirus type 2 (CPV-2) is a small, non-enveloped single stranded DNA virus that is classified within the family *Parvoviridae* in the species *Carnivore protoparvovirus 1* [1]. It is extremely stable in the environment; hence, environmental contamination and transmission via fomites are important in the epidemiology of the virus [2]. Canine parvovirus type 2 emerged as a novel pathogen in the late 1970s, most likely due to interspecies transmission of a closely related feline panleukopenia virus (FPV) [3,4,5]. Its emergence was associated with a pandemic of severe gastroenteritis in domestic dogs worldwide [6]. The virus evokes strong, long-lasting immunity following either infection or vaccination [7]. Hence, parvoviral enteritis is most commonly observed in young, unvaccinated dogs and continues to be an important cause of mortality in such dogs worldwide, including Sri Lanka [8,9].

The genome of CPV-2 is approximately 5.3 kbp long and comprises two main open reading frames (ORFs): ORF1 that codes for non-structural proteins (NSP) 1 and 2; and ORF2 that codes for structural viral proteins 1 and 2 (VP1 and VP2) [10]. Amino-acid substitutions at specific sites in the VP2 have been shown to affect antigenicity and the host-range of the virus and are the basis for the designation of CPV-2 subtypes [11,12,13]. 

The original CPV-2 was replaced by subtype CPV-2a (Gln-426 variant) soon after its emergence. By 1991, CPV-2b (Asp-426 variant) started to circulate concurrently with CPV-2a among dogs in many countries (Parrish, 1991). The most recent subtype, CPV-2c (Glu-426 variant) was first reported from Italy in 2001 [14]. It was later detected in Asia [15], USA [16], South America [17], Africa [18] and several other European countries [19]. The clinical significance of infections with different CPV-2 subtypes is poorly understood. Additionally, coinfections with multiple CPV-2 subtypes have been observed in domestic dogs [20,21]. 

Sri Lanka is a South Asian island located in the Indian Ocean. At 65,610 km^2^, Sri Lanka is the fourth largest island country in Asia with year-round tropical climate. The Palk Strait separates the island from mainland Asia by a 48 km wide ocean gap. Despite this geographical isolation, comparatively relaxed quarantine practices [22] hinder Sri Lankas’s ability to prevent introduction of animal pathogens. 

Reliable data on the Sri Lankan dog population are lacking. While some domestic dogs are restricted to the owner’s property (either as guard dogs or as pets), many owned dogs are allowed to roam the streets. Despite several ongoing control programs, the country also has a large population of stray dogs that are not associated with a household [23]. Most of the domestic cats in Sri Lanka are kept as outdoor cats. Free-roaming dogs and domestic cats are typically not vaccinated against any diseases other than rabies [24]. Additionally, Sri Lanka is home to several wild carnivores including the Sri Lankan golden jackal, Sri Lankan sloth bear and Sri Lankan leopard and another three species of small wild cats, five species of civets and four species of mongooses [25], all of which are potential hosts for carnivore protoparvoviruses. During the period of the study, 13 commercial canine parvovirus vaccines were registered in Sri Lanka [26]. Therefore, Sri Lanka provides a very favorable ecosystem for the rapid evolution of CPV-2.

The aim of the current study was to identify the CPV-2 subtypes currently circulating among domestic dogs and cats in Sri Lanka and to investigate the evolutionary patterns among selected Sri Lankan viruses in the context of contemporary global CPV-2 sequences.

## 2. Results

### 2.1. Sampled Animals

**Figure 1:**
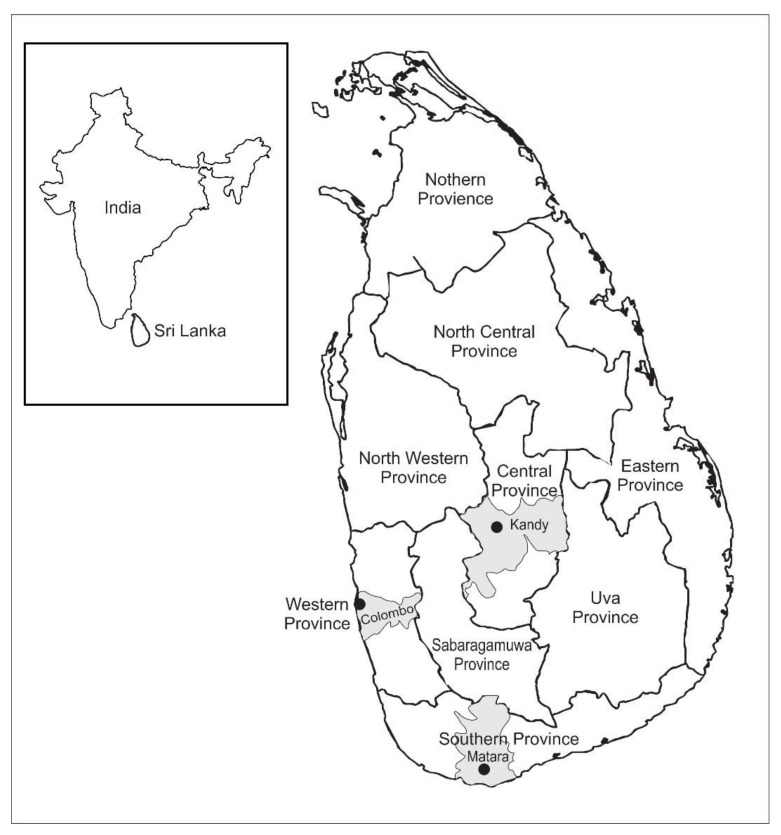
A map illustrating the administrative division in Sri Lanka and the location of the country across a narrow ocean gap from India (shown in the inset). Fecal samples from dogs and cats with diarrhea submitted to the Veterinary Microbiology laboratory at the University of Peradeniya (Kandy) from December 2015 to April 2017 from veterinary hospitals in Kandy (Central Province), Colombo (Western province) and Matara (Southern province) were used in the study.

Fecal samples from 50 domestic dogs and 13 domestic cats with suspected CPV-2 infection were included in the study (Table S1). The samples were from three geographically distinct provinces (Central, Western and Southern Provinces) in Sri Lanka (Figure 1). Most of the canine samples (30/50, 60%) and all feline samples (n = 13) were obtained from the Government Veterinary Hospital, Peradeniya in Kandy (Central Province). This hospital routinely serves Kandy and Matale districts and, occasionally, other nearby districts. The remaining canine samples originated from private practices in Colombo (Western Province, n = 7) and Matara (Southern Province, n = 13).

Most dogs were crossbreds (32/50, 64%), followed by German Shepherds (9/50, 18%), Rottweilers (3/50, 6%), Labrador Retrievers (2/50, 4%), Boxers, Pomeranians, Dachshunds and Dobermanns (1/50 each, 2%). The age was provided for 38 dogs and 11 cats. For these animals, the median age was 6 months (range 1–204) for dogs and 18 months (range 3–84) for cats. 

Only 16/50 (32%) dogs were reported to be vaccinated against CPV-2 at least once in the past, but the detailed vaccination history was often not available. The vaccination status of cats against feline panleukopenia virus was unknown.

### 2.2. CPV-2 PCR Positive Samples 

Two samples (#27 and #64) were negative in 18S RNA PCR and were excluded from the analysis. Of the remaining 61 samples, 40 (65.6%, 95% CI 53.0–76.2) tested positive for CPV-2 DNA by PCR with JS primers. This included 31/48 (64.6%, 95% CI 50.4–76.6) samples from dogs and 9/13 (69.2%, 95% CI 42.4–87.3) samples from cats. The remaining 21/61 (34.4%, 95% CI 23.7–46.9) samples were negative for CPV-2 DNA with both primer sets used. Altogether, 27/42 (64.3%, 95% CI 49.2–77.0) samples from the Government Hospital in Kandy, 7/13 (53.8%, 95% CI 29.1–76.8) samples from Matara in the Southern Province and 6/6 (100%, 95% CI 61.0–100%) samples from Colombo in the Western Province tested positive for CPV-2 DNA. 

Eight of fifteen (53.3%, 95% CI 30.1–75.2) dogs with a history of CPV-2 vaccination tested negative for CPV-2 DNA, but CPV-2 DNA was detected in samples from the remaining seven (46.7%, 95% CI 24.8–69.9) dogs (Table S1). These included two 2-month-old puppies and a 1-year-old dog that had been vaccinated a week prior to the development of diarrhea. The sample from one vaccinated dog (#27) was excluded from the analysis based on the negative 18SRNA PCR result. 

The majority (23/31, 74.2%) of CPV-2 positive dogs for which age was provided were ≤1 year of age. The median age for CPV-2 positive dogs was 5.5 months (range 1–204, n = 24), and the median age for CPV-2 positive cats was 18 months (range 4–36, n = 7).

### 2.3. CPV-2 Subtyping

Sri Lankan CPV-2 sequences represented all currently known subtypes (Table S1). Of the 40 CPV-2 positive samples, 20 (50%, 95% CI 35.2–64.8) were classified as CPV-2a, 8 (20%, 95% CI 10.5–34.7) as CPV-2b and 9 (22.5%, 95% CI 12.3–37.5) as CPV-2c. Two samples (5%, 95% CI 0.9–16.5) contained two different subtypes (CPV-2a and CPV-2c), and one sample (2.5%, 95% CI 0.9–12.9) was classified as CPV-2. All three CPV-2 subtypes were present in samples from Kandy, CPV-2a and CPV-2c in samples from Matara, while only CPV-2c was detected in samples from Colombo.

### 2.4. Haplotype Networks

**Figure 2:**
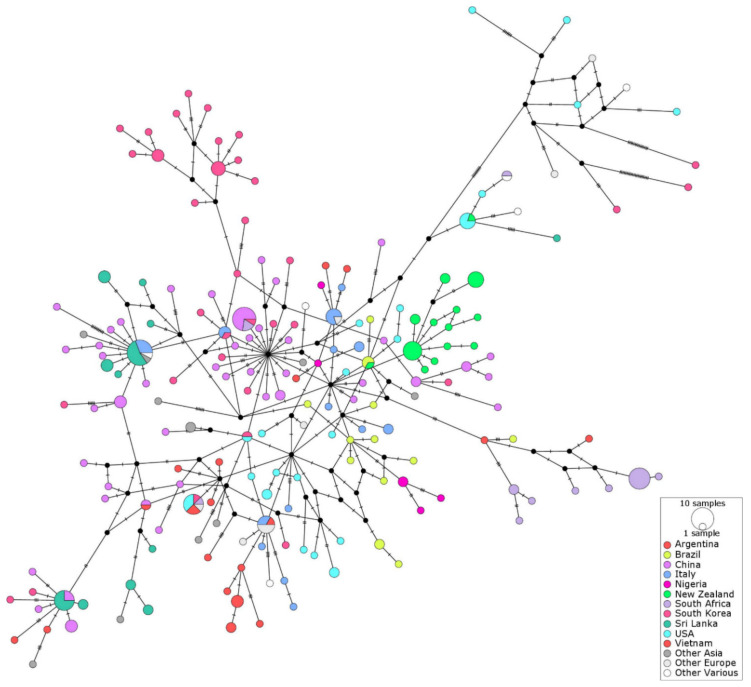
International haplotype network of CPV-2 viral protein 2 (VP2) sequences obtained from NCBI (n = 266) and those obtained in the current study (n = 31). The number of nucleotide substitutions between haplotypes is represented by ticks on branches. Nodes are scaled based on the number of representative sequences, and coloured based on the geographical area of origin. Inferred nodes are indicated with small closed black circles as they are not represented among sequences included in the network. Reticulation in a haplotype network indicates that the data did not contain appropriate signal to resolve a single pathway through a network, indicating that there is more than one possible sequence of mutations linking associated haplotypes. Sri Lankan sequences occupied at least three distinct regions of the network; hence, they were not monophyletic.

Median Joining haplotype network illustrating the genetic structure amongst 291 CPV-2 sequences based on 1381 bp of the VP2 gene (excluding gaps and unresolved sites) showed that Sri Lankan sequences occupied at least three distinct regions of the network; hence, they were not monophyletic (Figure 2). The distribution of CPV-2 haplotypes suggested that there was little barrier for CPV-2 movement between different geographical regions, with only 23% variability attributed to variation between populations (Table 1). 

**Figure 3:**
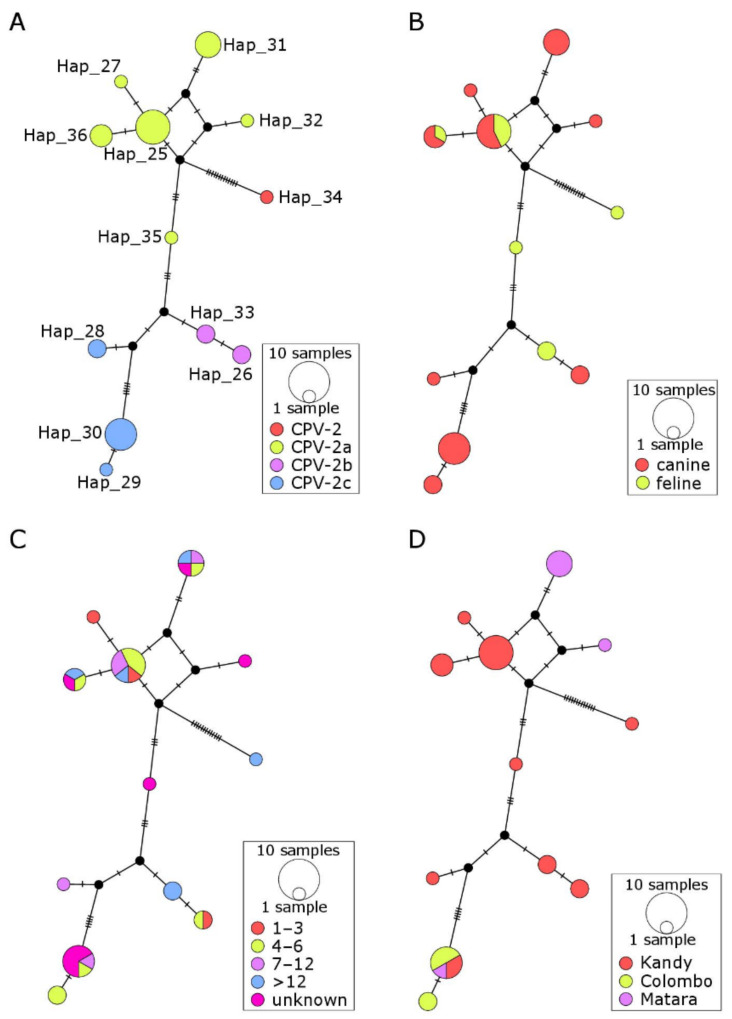
Median joining haplotype network illustrating the genetic structure amongst 31 canine parvovirus type 2 (CPV-2) sequences from Sri Lanka based on 1659 bp of the viral protein 2 (VP2). Nodes are scaled based on the number of representative sequences and colored based on the (**A**) CPV-2 subtype, (**B**) host, (**C**) age group (months) and (**D**) geographic location of the submitting veterinary clinic, as indicated in the legends. Inferred nodes are depicted as small closed black circles since they are not represented by the samples included in the analysis. Reticulation in a haplotype network indicates that the data did not contain appropriate signals to resolve a single pathway through the network, indicating that there is more than one possible sequence of mutation to reach a particular haplotype.

Median joining haplotype networks for characterizing the genetic structure amongst 31 Sri Lankan CPV-2 sequences did not correlate with the location of the veterinary clinic where the samples were submitted to the laboratory nor with the age group or type of the host from which the CPV-2 positive fecal samples were obtained (Figure 3, Table 1). There was, however, a clear structure in the network when samples were colored by the CPV-2 subtype (Figure 3A, Table 1).

## 3. Discussion

Canine parvovirus enteritis continues to be an important cause of morbidity and mortality among young dogs worldwide despite the availability of effective vaccines [27,28]. This appears to also be true for Sri Lanka, as CPV-2 was detected in feces from nearly 65% of diarrheic dogs tested in the current study. The majority of CPV-2 positive dogs were young, which is similar to results of a recent Sri Lankan based study [9] and to data from other countries [28,29,30]. Adult dogs are generally considered to be resistant to canine parvovirus enteritis, most likely due to long-lasting immunity acquired either from vaccination or prior infection [8]. As CPV-2 can survive in the environment for weeks to months [2], dogs are likely to be exposed to the virus throughout their lives, which may act as a booster of the pre-existing immunity. 

Although the vaccination data were available only for a small proportion of the sampled dogs, nearly half (7/16) of those reported as “vaccinated” were CPV-2 positive (Table S1). These cases do not necessarily represent vaccine failures, as reliable details of vaccinations (dates/types of vaccines used) were not provided, which renders it impossible to assess whether the dogs were vaccinated according to the current guidelines. Vaccinated puppies younger than 16 weeks of age that tested CPV-2 positive (e.g., puppies #15 and 16) may have been infected before the development of vaccine-induced immunity or during the incubation period. However, the lack of structure in the haplotype network colored by age indicates that none of the genotypes were more likely to infect older dogs than puppies up to 6 months of age. This suggests that it is unlikely that CPV-2 of any particular genotype was more likely to cause disease in older dogs that may be expected to be partially immune either through prior vaccination or exposure to the field virus. 

**Figure 4:**
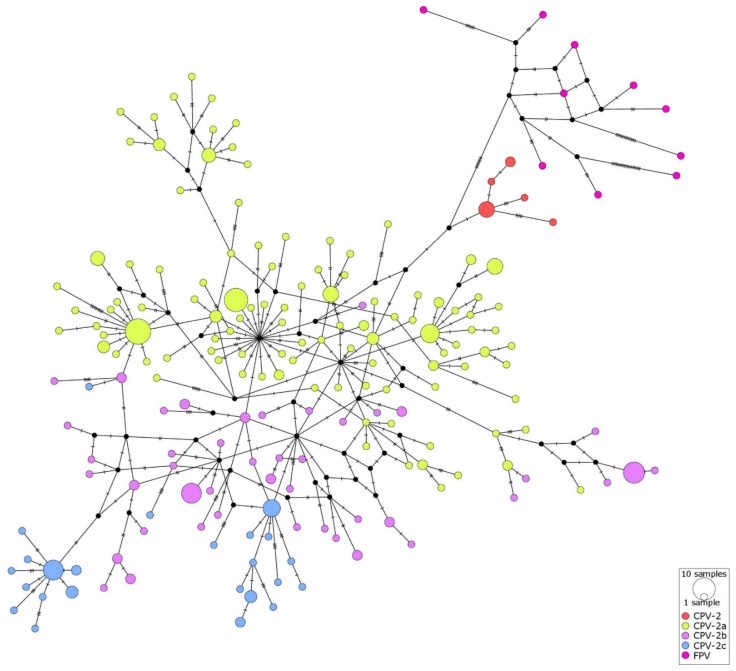
International haplotype network of CPV-2 viral protein 2 (VP2) sequences obtained from NCBI (n = 266) and those obtained in the current study (n = 31). The number of nucleotide substitutions between haplotypes is represented by ticks on branches. Nodes are scaled based on the number of representative sequences and coloured based on the CPV-2 subtype including feline panleukopenia virus (FPV). Inferred nodes are indicated with small closed black circles as they are not represented among sequences included in the network. Reticulation in a haplotype network indicates that the data did not contain appropriate signals to resolve a single pathway through a network, indicating that there is more than one possible sequence of mutation linking the associated haplotypes.

Limited genetic structure observed for CPV-2 sequences obtained from the same geographical location (Kandy, Matara, Colombo districts; Figure 3D) indicates that there were no strong barriers for CPV-2 spread between different geographical regions of Sri Lanka. This is supported by the clear structure in the network colored by a CPV-2 subtype (Figure 3A, Table 1), indicating that genetically similar CPV-2 sequences were mostly related to one another; hence, their detection in various parts of the country was likely to reflect the movement of the virus rather than independent local evolution. This is not surprising considering the frequent movement of people between districts and the extreme stability of CPV-2, with high potential for spread via fomites. The international network data (Figure 4) suggest that CPV-2 may also be moving relatively unhindered between different countries, which is indicated by the presence of multiple clusters comprising sequences of different geographical origin and supported by AMOVA results (Table 1). 

Sri Lankan CPV-2 sequences were not monophyletic, with several sequences (shown in dark green in Figure 2) present in three different areas of the international network mixed with sequences from other countries/regions. This was dissimilar to the largely monophyletic group of CPV-2 sequences from New Zealand (shown in bright green in Figure 2), as has been reported previously [28]. Both New Zealand and Sri Lanka are small island countries, and hence the data from Sri Lanka were expected to be more comparable to the data from New Zealand than to the data from other geographical regions represented in the network. Results of the international haplotype network suggest that despite the geographical similarities between Sri Lanka and New Zealand, the epidemiology of CPV-2 differs between the two countries. This is unlikely to be explained by the differences in the timing of sample collection, as both Sri Lankan and New Zealand sequences were collected within a period of approximately one year. In addition, the decade of sample collection was not correlated with the genetic structure in the international network (Table 1), which was in agreement with the previous report that used a smaller dataset [28]. One possible explanation for these findings is the less stringent border control in Sri Lanka in comparison to the very strict border security in New Zealand. This may facilitate a more frequent introduction of CPV-2 from overseas to Sri Lanka than to New Zealand. Alternatively, the variability of CPV-2 sequences in Sri Lanka may reflect the ongoing evolution of the locally circulating viruses. The use of a larger number of different CPV-2 vaccines in Sri Lanka (n = 13) in comparison to New Zealand (n = 3) may have driven more dynamic evolution of CPV-2 in Sri Lanka as compared to New Zealand despite the fact that vaccines used in both countries are based predominantly or exclusively on the original CPV-2. It has also been suggested that wild carnivores such as raccoons may have played a role in the evolution of CPV-2 [31]. While neither Sri Lanka nor New Zealand has an endemic population of raccoons, a number of other carnivore species are susceptible to infection with carnivore protoparvoviruses [32], and hence may have played a role in CPV-2 evolution. It could be hypothesized that the higher numbers and diversity of wild carnivores living in Sri Lanka in comparison to the very limited number of wild carnivore species in New Zealand may have contributed to the relative genetic stability of CPV-2 in New Zealand compared to Sri Lanka.

Although all three CPV-2 subtypes were identified in the current study, CPV-2a was detected most frequently. The Sri Lankan CPV-2a showed the amino acid changes that originally differentiated CPV-2a from the CPV-2 at positions 87 (Met → Leu), 101 (Ile → Thre), 300 (Gly → Ala) and 305 (Tyr → Asp), but all lacked the Val →Ile mutation at position 555 that was present in early CPV-2a [11,12,33]. This reversion to the original CPV-2 sequence at position 555 has been reported in CPV-2a from several other countries, including New Zealand [28], Australia [27], Italy [34], Brazil [35] and Thailand [36], and was maintained in the later antigenic subtypes CPV-2b and CPV-2c. The relatively high frequency of CPV-2a detection in the current study was similar to the situation in New Zealand [28] or Thailand [36] but in contrast to results recently reported from Sri Lanka by another group [9]. In the latter study, 12/20 (60%) of CPV-2 were subtyped as CPV-2c and 7/20 (35%) as CPV-2a, with only one CPV-2b. As all the viral sequences in that study were derived from vaccinated dogs, the authors suggested that CPV-2c may be more likely than other subtypes to escape protection offered by vaccination [9]. While we cannot fully exclude this possibility because vaccination history was not available for any of the CPV-2c positive dogs in the current study, both CPV-2a and CPV-2b were detected in samples from vaccinated dogs, which does not seem to support such views. It does, however, highlight the dynamic state of the local CPV-2 evolution, which can be further illustrated by the detection of only CPV-2c viruses from the Colombo district in the current study or a recent replacement of CPV-2a with CPV-2b and introduction of CPV-2c in Australia [27,37]. 

An alternative explanation for the predominance of CPV-2a among sampled animals is that CPV-2a was most virulent, as only clinically affected dogs were sampled in the current study. If less virulent CPV-2 of different genotypes circulated among dogs in Sri Lanka without causing clinical disease, we would not have detected these. Such possibility has been suggested previously [27,28], but the change of the make-up of genotypes within a given region over time, as demonstrated using a similar sampling strategies, e.g., in Australia [38,39] or in Italy [8], would argue against this suggestion. Although some authors attempted to make an association between the CPV-2 subtype and the severity of disease [39,40], this is inherently difficult to demonstrate because of the confounding effects of the host-factors such as age, breed or the level of immunity on the outcome of CPV-2 infection [28,29,41]. Cross-sectional sampling of young dogs with and without apparent gastrointestinal disease in CPV-2 endemic areas would be needed to elucidate if certain genotypes are associated with more severe clinical diseases than others.

There was no structure present in the Sri Lankan network colored by the host (feline versus canine). This suggests that the locally circulating viruses can infect both dogs and cats, and that cats may potentially contribute to the spread of CPV-2. Similar conclusions have been recently reached by the researchers from Thailand [36], which suggests a potential role of free-roaming cats in transmission of CPV-2 to dogs. It remains unknown how prevalent CPV-2 infection is among cats and how many of those infections are asymptomatic. The extent of protection against CPV-2 infection and shedding offered by the currently used FPV vaccines is also unknown. These questions should be addressed in future studies as such knowledge would be important for the implementation of appropriate control measures against CPV-2-associated diseases in both dogs and cats. Interestingly, none of the cats tested was positive for FPV. Unfortunately, the vaccination history for any of the CPV-2 positive cats included in the current study was unknown. However, considering the generally low rate of cat vaccination in Sri Lanka, these cats were more likely to be unvaccinated than vaccinated against FPV.

One sample from a cat was subtyped as the original CPV-2. This was an unexpected result, as the original CPV-2 was believed not to be infectious for cats. In addition, it circulated among dogs only for a short period of time after the initial cross-species transmission in the late 1980s [3]. It has been, however, extensively used in CPV-2 vaccines. For this reason, the detection of CPV-2 from the fecal sample of a cat may represent a mistake made at some point between sample collection and subtyping. Alternatively, this particular virus was able to replicate, to some extent, in the cat after a (presumably) parenteral administration of a CPV-2-based vaccine.

The limitations of the study include the small sample size and lack of reliable vaccination data for the majority of dogs sampled, which precluded any definite conclusions regarding the existence of a possible vaccine-escape mutant to be made. The data from the international network should also be interpreted with caution due to an inevitable bias introduced by the fact that the GenBank database is based on voluntarily submitted sequences. As such, not all regions/decades were represented equally well within the network. Finally, an opportunistic sampling strategy employed in the study precludes extrapolation of the frequency of CPV-2 variant detection to other canine or feline populations in Sri Lanka. 

In conclusion, we have generated and analyzed the largest dataset of contemporary CPV-2 sequences from a selected population of dogs and cats in Sri Lanka. Our data have shown that there is considerable variability of CPV-2 genotypes circulating in Sri Lanka. Both local evolution and introduction from other countries are likely to have contributed to this variability. The differences between epidemiology of the virus in Sri Lanka and a similar island country such as New Zealand suggest the possibility that the local evolution of CPV-2 may be facilitated by the presence of wild carnivores, but this hypothesis needs to be tested in further studies. The similarity of CPV-2 obtained from cats and dogs suggests that cats may play a role in the spread of CPV-2. Hence, the routine vaccination of cats with core feline antigens (including FPV) may facilitate control measures for canine parvovirus enteritis, but the extent of protection against carnivore parvoviruses other than FPV needs to be elucidated in future studies. 

## 4. Materials and Methods

### 4.1. Sources of Samples

Fecal samples from domestic dogs and cats with diarrhea received by the Veterinary Microbiology laboratory at the University of Peradeniya from December 2015 to April 2017 were used in this study. The samples for which “CPV-2” was listed as one of the differential diagnoses by the referring veterinarians were eligible for inclusion. Each fecal sample was sent to the laboratory within 24 h of collection on ice packs and was accompanied by a submission letter that included brief signalment and vaccination details (if available) for the dog or the cat. 

### 4.2. Processing of Samples

The samples were either processed immediately after arrival at the laboratory or stored at −80 °C for later processing. A small amount of the fecal material (100–200 µL) was mixed with phosphate buffered saline pH 7.0 at 1:3 ratio just before DNA extraction. Total genomic DNA was extracted using the QIAamp^®^ DNA Stool Mini Kit (Qiagen, Hilden, Germany), according to the manufacturer’s instructions. Extracted DNA was stored at −80 °C and shipped on dry ice to Massey University, New Zealand, for CPV-2 PCR and subtyping. 

### 4.3. CPV-2 PCR

Conventional PCR reaction targeting a VP2 gene using published primers VP2.JS1.F and VP2.JS2.R [27,28] was used to amplify a 1975 bp fragment of CPV-2 DNA. Each PCR reaction consisted of 0.4 µM of each primer and 1 µL DNA template in a total volume of 20 µL using HotFirePol master mix with the final concentration of 2.5 mM Mg (Solis BioDyne, Tartu, Estonia). The cycling conditions included an initial denaturation at 95 °C for 15 min, followed by 40 cycles of denaturation (95 °C for 15 s), annealing (52 °C for 10 s) and extension (72 °C for 2 min), with the final extension step at 72 °C for 7 min and 4 °C hold. DNA from a contemporary CPV-2 positive sample (confirmed by sequencing) and water were included with every PCR run as positive and negative controls, respectively. PCR products were subjected to electrophoresis through a 1% agarose gel (Axygen, Corning, NY, USA) containing 0.5 µg/mL ethidium bromide in Tris-Acetate-EDTA (TAE) buffer for 90 min at 90 V and visualised using GelDoc reader (Bio-Rad Laboratories, Hercules, CA, USA). The test was considered valid if positive and negative controls produced expected results. DNA was extracted from bands of the expected size and submitted for sequencing to the Massey Genome Centre (Massey University, Palmerston North, New Zealand).

Samples that tested negative with JS primers were retested with another set of CPV-2 specific primers (CPV555) designed to amplify a shorter (582 bp) PCR product [14]. The cycling conditions included an initial denaturation at 95 °C for 15 min, followed by 40 cycles of denaturation (95 °C for 10 s), annealing (52 °C for 10 s) and extension (72 °C for 1 min), with the final extension step at 72 °C for 5 min and 4 °C hold. Samples that were negative in PCR with both JS and CPV555 primers were tested with primers complementary to the 18S ribosomal RNA gene in order to check for the presence of amplifiable DNA [42].

### 4.4. CPV Subtyping

Each long CPV-2 PCR product was sequenced from four sequencing primers (VP2.JS1.F, VP2.JS2.R, VP2.JS3.F and VP2.JS3.R), trimmed to exclude low quality ends and mapped to a reference sequence (GenBank accession number M38245.1) using a bioinformatics software (Geneious Pro 9.1.8, Biomatters Ltd., 2009, Auckland, New Zealand) [43]. If a shorter PCR product was available, it was also sequenced from both forward and reverse CPV555 primers and added to the assembly. The viruses were assigned to specific CPV-2 subtypes based on the presence of selected amino-acid residues at specified positions (Table 2). 

If the sequence at the defining amino acid position 426 was poorly resolved, the PCR product was cloned using the TOPO TA cloning kit for sequencing with TOP-10 chemically competent *E. coli* (Thermo Fisher Scientific, Waltham, MA, USA), according to the manufacturer’s instructions. The CPV-2 subtype was determined by sequencing five recombinant *E. coli* colonies. 

### 4.5. Molecular Network Analyses

A consensus CPV-2 sequence was generated from each assembly and manually curated. A total of 31 consensus CPV-2 sequences that were at least 1.6 kbp in length with less than 160 bp of internal gaps were used for the network analyses. These included sequences from 23 dogs and 8 cats. Additional CPV-2 VP2 sequences (n = 266) originating from various countries were obtained from the National Centre for Biotechnology Information (NCBI) database (Table S2). Only CPV-2 sequences that span the entire region of consensus Sri Lankan CPV-2 sequences were eligible for inclusion in the analysis. 

All 297 nucleotide sequences were aligned using MUSCLE alignment within Geneious, exported to MEGAX [44] and saved as a nexus file, which was then used as an input file to generate a haplotype list in the DnaSP software (available from http://www.ub.edu/dnasp/ (accessed on 27 August 2021)). Sites with gaps or missing/ambiguous data were excluded. The sequences representative of the haplotypes were then realigned in Geneious for both the international network (190 haplotypes) and for the Sri Lanka only network (15 haplotypes) and then exported in nexus format with trait blocks added to represent geographical region, decade of isolation and subtype. For the Sri Lankan only network, additional trait blocks were added to represent the age groups of the sampled animals, species (feline or canine) and geographical location (Kandy, Colombo, Matara) of the veterinary clinic where the samples were submitted. The median joining haplotype networks were drawn using default parameters in PopART version 1.6 [45]. Analysis of molecular variance (AMOVA within PopART) was used to test for correlation between population genetic structure of the CPV-2 sequences and selected traits. The strength of correlation was represented by a PhiPT value, with 0 indicating no correlation and 1 indicating perfect correlation. The corresponding *p* values were generated by reference to 1000 random permutations of the input data.

## Figures and Tables

**Table 1:** Analysis of molecular variance (AMOVA) results indicating the strength of correlation between population genetic structure and selected traits based on partial sequence of viral protein 2 of canine parvovirus type 2 (CPV-2).

Test	Variation within Populations	Variation between Populations	PhiST	*p*
International network				
Geographical region ^1^	77.1%	22.9%	0.23	<0.001
Decade of collection ^2^	83.8%	16.2%	0.16	<0.001
Subtype ^3^	51.7%	48.3%	0.48	<0.001
Sri Lankan network				
Location ^4^	76.5%	23.5%	0.23	0.001
Age-group ^5^	106.2%	−6.2%	−0.06	0.879
Host ^6^	95.6%	4.4%	0.04	0.080
Subtypes ^7^	16.6%	83.4%	0.83	<0.001

^1^ Argentina; Brazil; China; Italy; Nigeria; New Zealand; South Africa; South Korea; Sri Lanka; USA; Vietnam; Other Asia; Other Europe; Other Various. ^2^ 1960–1969; 1970–1979; 1980–1989; 1990–1999; 2000–2009; 2010–2019; unknown. ^3^ CPV-2; CPV-2a; CPV-2b; CPV-2c; feline panleukopenia virus. ^4^ Kandy; Colombo; Matara. ^5^ 1–3; 4–6; 7–12; >13 months; unknown. ^6^ Canine; feline. ^7^ CPV-2; CPV-2a; CPV-2b; CPV-2c.

**Table 2:** Canine parvovirus type 2 (CPV-2) subtype designation based on nucleotide and deduced amino acid substitutions at selected positions within sequences encoding viral protein 2 (VP2).

Subtype	Nucleotide (Amino Acid) Position ^1^					
	3045 (87)	3087 (101)	3684 (300)	3699 (305)	4062 (426)	4449 (555)
CPV-2	ATG (Met)	ATT (Ile)	GCT (Ala)	GAT (Asp)	AAT (Asn)	GTA (Val)
CPV2a	TTG (Leu)	ACT (Thr)	GGT (Gly)	TAT (Tyr)	AAT (Asn)	ATA (Ile)
CPV2b	TTG (Leu)	ACT (Thr)	GGT (Gly)	TAT (Tyr)	GAT (Asp)	ATA (Ile)
CPV2c	TTG (Leu)	ACT (Thr)	GGT (Gly)	TAT (Tyr)	GAA (Glu)	ATA (Ile)

^1^ Position of the first nucleotide in a codon (deduced amino acid position) based on the reference sequence (GenBank accession no. M38245).

## Data Availability

The Sri Lankan CPV-2 sequences obtained in this study have been deposited in GenBank under accession numbers MZ398072–MZ398102.

## Supplementary Materials

The following are available online at https://www.mdpi.com/article/10.3390/pathogens10091102/s1, Table S1: Signalment, vaccination data and results for canine parvovirus type 2 (CPV-2) testing of fecal samples (n = 63) collected from dogs (n = 50) and cats (n = 13) via three different veterinary clinics in Sri Lanka between December 2015 to April 2017, Table S2: Canine parvovirus type 2 sequences included in the international network.

## References

[1] Cotmore S.F., Agbandje-McKenna M., Canuti M., Chiorini J.A., Eis-Hubinger A.M., Hughes J., Mietzsch M., Modha S., Ogliastro M., Penzes J.J. (2019). ICTV Virus Taxonomy Profile: Parvoviridae. J. Gen. Virol..

[2] Gordon J.C., Angrick E.J. (1986). Canine parvovirus: Environmental effects on infectivity. Am. J. Vet. Res..

[3] Carmichael L.E. (2005). An annotated historical account of canine parvovirus. J. Vet. Med. B Infect. Dis. Vet. Public Health.

[4] Parrish C.R., Holmes E.C., Morens D.M., Park E.C., Burke D.S., Calisher C.H., Laughlin C.A., Saif L.J., Daszak P. (2008). Cross-species virus transmission and the emergence of new epidemic diseases. Microbiol. Mol. Biol. Rev..

[5] Wilson N.D. (1980). Origin of canine parvovirus. Vet. Rec..

[6] Hoelzer K., Parrish C.R. (2010). The emergence of parvoviruses of carnivores. Vet. Res..

[7] Schultz R.D., Thiel B., Mukhtar E., Sharp P., Larson L.J. (2010). Age and long-term protective immunity in dogs and cats. J. Comp. Pathol..

[8] Decaro N., Buonavoglia C. (2012). Canine parvovirus–a review of epidemiological and diagnostic aspects, with emphasis on type 2c. Vet. Microbiol..

[9] Gamage B., Dissanayake D.R.A., Prasada D.V.P., Silva I.D. (2020). Risk, prognosis and causality of parvo viral enteritis in dogs in Sri Lanka. Comp. Immunol. Microbiol. Infect. Dis..

[10] Reed A.P., Jones E.V., Miller T.J. (1988). Nucleotide sequence and genome organization of canine parvovirus. J. Virol..

[11] Parrish C.R., Aquadro C.F., Strassheim M.L., Evermann J.F., Sgro J.Y., Mohammed H.O. (1991). Rapid antigenic-type replacement and DNA sequence evolution of canine parvovirus. J. Virol..

[12] Parrish C.R. (1999). Host range relationships and the evolution of canine parvovirus. Vet. Microbiol..

[13] Hueffer K., Parrish C.R. (2003). Parvovirus host range, cell tropism and evolution. Curr. Opin. Microbiol..

[14] Buonavoglia C., Martella V., Pratelli A., Tempesta M., Cavalli A., Buonavoglia D., Bozzo G., Elia G., Decaro N., Carmichael L. (2001). Evidence for evolution of canine parvovirus type 2 in Italy. J. Gen. Virol..

[15] Nakamura M., Tohya Y., Miyazawa T., Mochizuki M., Phung H.T., Nguyen N.H., Huynh L.M., Nguyen L.T., Nguyen P.N., Nguyen P.V. (2004). A novel antigenic variant of Canine parvovirus from a Vietnamese dog. Arch. Virol..

[16] Hong C., Decaro N., Desario C., Tanner P., Pardo M.C., Sanchez S., Buonavoglia C., Saliki J.T. (2007). Occurrence of canine parvovirus type 2c in the United States. J. Vet. Diagn. Investig..

[17] Perez R., Francia L., Romero V., Maya L., Lopez I., Hernandez M. (2007). First detection of canine parvovirus type 2c in South America. Vet. Microbiol..

[18] Touihri L., Bouzid I., Daoud R., Desario C., El Goulli A.F., Decaro N., Ghorbel A., Buonavoglia C., Bahloul C. (2009). Molecular characterization of canine parvovirus-2 variants circulating in Tunisia. Virus Genes.

[19] Decaro N., Desario C., Addie D.D., Martella V., Vieira M.J., Elia G., Zicola A., Davis C., Thompson G., Thiry E. (2007). The study molecular epidemiology of canine parvovirus, Europe. Emerg. Infect. Dis..

[20] Miranda C., Carvalheira J., Parrish C.R., Thompson G. (2015). Factors affecting the occurrence of canine parvovirus in dogs. Vet. Microbiol..

[21] Miranda C., Thompson G. (2016). Canine parvovirus: The worldwide occurrence of antigenic variants. J. Gen. Virol..

[22] Import/Export–Procedure for Import/Export. http://www.daph.gov.lk/web/index.php?option=com_content&view=article&id=24&Itemid=126&lang=en.

[23] Ubeyratne K., Srikitjakarn L., Pfeiffer D., Kong F., Sunil-Chandra N., Chaisowwong W., Hemwan P. (2020). A Knowledge, Attitudes and Practices (KAP) survey on canine rabies prevention and control in four rural areas of Sri Lanka. Transbound. Emerg. Dis..

[24] Sanchez-Soriano C., Gibson A.D., Gamble L., Burdon Bailey J.L., Green S., Green M., Bronsvoort B.M.D., Handel I.G., Mellanby R.J., Mazeri S. (2019). Development of a high number, high coverage dog rabies vaccination programme in Sri Lanka. BMC Infect. Dis..

[25] Yapa A., Ratnavira G. (2013). The Mammals of Sri Lanka.

[26] Veterinary Drug Control Authority of Sri Lanka (2021). Registered Veterinary Medical Products for Import and Free Sale. http://www.daph.gov.lk/web/images/content_image/vra/2021/VDCAregisteredVMPs_for_import_and_free_sale_updated_to_31.12.2020.pdf.

[27] Meers J., Kyaw-Tanner M., Bensink Z., Zwijnenberg R. (2007). Genetic analysis of canine parvovirus from dogs in Australia. Aust. Vet. J..

[28] Ohneiser S.A., Hills S.F., Cave N.J., Passmore D., Dunowska M. (2015). Canine parvoviruses in New Zealand form a monophyletic group distinct from the viruses circulating in other parts of the world. Vet. Microbiol..

[29] Houston D.M., Ribble C.S., Head L.L. (1996). Risk factors associated with parvovirus enteritis in dogs: 283 cases (1982–1991). J. Am. Vet. Med. Assoc..

[30] Giraldo-Ramirez S., Rendon-Marin S., Ruiz-Saenz J. (2020). Phylogenetic, Evolutionary and Structural Analysis of Canine Parvovirus (CPV-2) Antigenic Variants Circulating in Colombia. Viruses.

[31] Allison A.B., Harbison C.E., Pagan I., Stucker K.M., Kaelber J.T., Brown J.D., Ruder M.G., Keel M.K., Dubovi E.J., Holmes E.C. (2012). Role of multiple hosts in the cross-species transmission and emergence of a pandemic parvovirus. J. Virol..

[32] Steinel A., Parrish C.R., Bloom M.E., Truyen U. (2001). Parvovirus infections in wild carnivores. J. Wildl. Dis..

[33] Truyen U., Evermann J.F., Vieler E., Parrish C.R. (1996). Evolution of canine parvovirus involved loss and gain of feline host range. Virology.

[34] Martella V., Decaro N., Elia G., Buonavoglia C. (2005). Surveillance activity for canine parvovirus in Italy. J. Vet. Med. B Infect. Dis. Vet. Public Health.

[35] Castro T.X., Costa E.M., Leite J.P., Labarthe N.V., Cubel Garcia R.C. (2011). Monitoring of canine parvovirus (CPV) strains detected in vaccinated puppies in Brazil. Res. Vet. Sci..

[36] Inthong N., Kaewmongkol S., Meekhanon N., Sirinarumitr K., Sirinarumitr T. (2020). Dynamic evolution of canine parvovirus in Thailand. Vet. World.

[37] Clark N.J., Seddon J.M., Kyaw-Tanner M., Al-Alawneh J., Harper G., McDonagh P., Meers J. (2018). Emergence of canine parvovirus subtype 2b (CPV-2b) infections in Australian dogs. Infect. Genet. Evol..

[38] Woolford L., Crocker P., Bobrowski H., Baker T., Hemmatzadeh F. (2017). Detection of the Canine Parvovirus 2c Subtype in Australian Dogs. Viral. Immunol..

[39] Franzo G., Tucciarone C.M., Casagrande S., Caldin M., Cortey M., Furlanello T., Legnardi M., Cecchinato M., Drigo M. (2019). Canine parvovirus (CPV) phylogeny is associated with disease severity. Sci. Rep..

[40] Moon H.-S., Lee S.-A., Lee S.-G., Choi S.-Y., Kim D., Hyun C. (2008). Comparison of the pathogenicity in three different Korean canine parvovirus 2 (CPV-2) isolates. Vet. Microbiol..

[41] Kalli I., Leontides L.S., Mylonakis M.E., Adamama-Moraitou K., Rallis T., Koutinas A.F. (2010). Factors affecting the occurrence, duration of hospitalization and final outcome in canine parvovirus infection. Res. Vet. Sci..

[42] Schmittgen T.D., Zakrajsek B.A. (2000). Effect of experimental treatment on housekeeping gene expression: Validation by real-time, quantitative RT-PCR. J. Biochem. Biophys. Methods.

[43] Kearse M., Moir R., Wilson A., Stones-Havas S., Cheung M., Sturrock S., Buxton S., Cooper A., Markowitz S., Duran C. (2012). Geneious Basic: An integrated and extendable desktop software platform for the organization and analysis of sequence data. Bioinformatics.

[44] Kumar S., Stecher G., Li M., Knyaz C., Tamura K. (2018). MEGA X: Molecular Evolutionary Genetics Analysis across Computing Platforms. Mol. Biol. Evol..

[45] Leigh J., Bryant D. (2015). Popart: Full-feature software for haplotype network construction. Methods Ecol. Evol..

