# Supplementary material for: Genomic Variability of Canine Parvoviruses from a Selected Population of Dogs and Cats in Sri Lanka
Source: Pathogens. 2021 Aug 29;10(9):1102. doi: 10.3390/pathogens10091102 (PMC8466987; doi:10.3390/pathogens10091102)
Supplement: Supplementary file 1 [file pathogens-10-01102-s001.zip › pathogens-1317941-supplementary.pdf]

Table 1. Signalment, vaccination data and results for canine parvovirus type 2 (CPV-2) testing of fecal samples (n=63) collected from dogs (n=50) and cats (n=13) via three different veterinary clinics in Sri Lanka between December 2015 to April 2017.

ID	Breed	Sex	Age (months)	Vaccination	Time since last vaccination (months)	Province	CPV-2 (pos/neg)	CPV-2 sub-type
1	Xbred	F	1	UNK	UNK	Central	neg	
2	Xbred	M	7	UNK	UNK	Central	pos	CPV-2a
3	Xbred	M	6	UNK	UNK	Central	pos	CPV-2b
4	Xbred	M	204	UNK	UNK	Central	pos	CPV-2b
5	Xbred	M	12	yes	13	Central	pos	CPV-2a
6	Xbred	M	3	UNK	UNK	Central	pos	CPV-2a
7	Pomeranian	M	10	yes	7	Central	neg	
8	Xbred	M	60	yes	45	Central	neg	
9	Rottweiler	M	12	yes	10	Central	pos	CPV-2b
10	GSD	F	42	yes	4	Central	neg	
11	Labrador	F	38	yes	11	Central	neg	
12	GSD	M	6	yes	3	Central	pos	CPV-2a
13	GSD	M	14	yes	12	Central	neg	
14	GSD	F	3	yes	1	Central	neg	
15	Xbred	F	2	yes	UNK	Central	pos	CPV-2a
16	Labrador	M	2	yes	1	Central	pos	CPV-2b
17	GSD	M	18	yes	0.3	Central	pos	CPV-2b
18	GSD	M	12	yes	UNK	Central	pos	CPV-2b
19	Xbred	M	4	UNK	UNK	Central	pos	CPV-2a
20	Boxer	F	2	UNK	UNK	Central	pos	CPV-2c
21	Dachshund	F	96	yes	> 12	Central	neg	
22	Xbred	F	24	yes	1	Central	neg	
25	Xbred	M	5	UNK	UNK	Western	pos	CPV-2c
26	Xbred	M	5	UNK	UNK	Western	pos	CPV-2c
27	Xbred	M	48	yes	46	Western	neg[1]	
28	Xbred	M	8	UNK	UNK	Western	pos	CPV-2c
29	Xbred	M	4	UNK	UNK	Western	pos	CPV-2c
30	Xbred	-	UNK	UNK	UNK	Western	pos	CPV-2c
31	Xbred	UNK	UNK	UNK	UNK	Western	pos	CPV-2c
32	GSD	M	6	UNK	UNK	Southern	neg	
33	GSD	M	14	UNK	UNK	Southern	neg	
34	Xbred	UNK	108	UNK	UNK	Southern	pos	CPV-2a
35	Xbred	UNK	UNK	UNK	UNK	Southern	neg	
36	Xbred	UNK	UNK	UNK	UNK	Southern	neg	
37	Xbred	UNK	UNK	UNK	UNK	Southern	neg	
38	GSD	UNK	5	UNK	UNK	Southern	pos	CPV-2a
39	Xbred	UNK	5	UNK	UNK	Southern	pos	CPV-2a
40	Xbred	UNK	7	UNK	UNK	Southern	pos	CPV-2a
41	Xbred	UNK	UNK	UNK	UNK	Southern	pos	CPV-2a
42	Xbred	UNK	UNK	UNK	UNK	Southern	pos	CPV-2a CPV-2c
43	Xbred	UNK	UNK	UNK	UNK	Southern	pos	CPV-2c
44	Xbred	UNK	UNK	UNK	UNK	Southern	neg	
54	Rottweiler	UNK	1	UNK	UNK	Central	pos	CPV-2a
55	Rottweiler	F	17	UNK	UNK	Central	pos	CPV-2a
56	Dobermann	UNK	UNK	UNK	UNK	Central	pos	CPV-2a CPV-2c
58	Xbred	UNK	UNK	UNK	UNK	Central	pos	CPV-2c
61	Xbred	F	5	UNK	UNK	Central	pos	CPV-2a

63	Xbred	UNK	UNK	UNK	UNK	Central	neg	
64	Xbred	UNK	UNK	UNK	UNK	Central	neg[1]	
65	Xbred	UNK	UNK	UNK	UNK	Central	neg	
Cats								
45	Xbred	M	24	UNK	UNK	Central	pos	CPV-2a
46	Xbred	UNK	18	UNK	UNK	Central	pos	CPV-2b
47	Xbred	F	28	UNK	UNK	Central	pos	CPV-2
48	Xbred	F	36	UNK	UNK	Central	pos	CPV-2b
49	Xbred	UNK	4	UNK	UNK	Central	pos	CPV-2a
50	Xbred	UNK	5	UNK	UNK	Central	pos	CPV-2a
51	Xbred	F	5	UNK	UNK	Central	neg	
52	Xbred	M	UNK	UNK	UNK	Central	pos	CPV-2a
53	Xbred	UNK	60	UNK	UNK	Central	neg	
57	Xbred	UNK	UNK	UNK	UNK	Central	pos	CPV-2a
59	Xbred	F	84	UNK	UNK	Central	neg	
60	Persian	UNK	12	UNK	UNK	Central	pos	CPV-2a
62	Xbred	F	3	UNK	UNK	Central	neg	

[1] No amplifiable DNA present: sample excluded from the study. Abbreviations: unknown (UNK); male (M); female (F); crossbred (Xbred); positive (pos); negative (neg).

Table 2. Canine parvovirus type 2 sequences included in the international network.

No	Country of Origin	Isolate/strain	Accession Number	Subtype	Year	Haplotype
1	Argentina	Arg9	JF346754	CPV-2a	2003	Hap_130
2	Argentina	Arg5	JF414817	CPV-2b	2003	Hap_131
3	Argentina	Arg32	JF414818	CPV-2c	2008	Hap_132
4	Argentina	Arg35	JF414819	CPV-2c	2008	Hap_18
5	Argentina	Arg44	JF414820	CPV-2c	2003	Hap_133
6	Argentina	Arg48	JF414821	CPV-2c	2009	Hap_134
7	Argentina	Arg64	JF414822	CPV-2c	2010	Hap_134
8	Argentina	Arg60	JF414823	CPV-2c	2009	Hap_134
9	Argentina	Arg66	JF414824	CPV-2c	2010	Hap_135
10	Argentina	Arg67	JF414825	CPV-2c	2010	Hap_135
11	Argentina	Arg68	JF414826	CPV-2c	2010	Hap_136
12	Australia	FPV-193	X55115	FPV	1970	Hap_190
13	Brazil	BR6-80	DQ340404	CPV-2a	1980	Hap_40
14	Brazil	BR137-80	DQ340406	CPV-2a	1980	Hap_40
15	Brazil	BR183-85	DQ340409	CPV-2a	1985	Hap_41
16	Brazil	BR315-86	DQ340410	CPV-2a	1986	Hap_42
17	Brazil	BR8-90	DQ340411	CPV-2a	1990	Hap_43
18	Brazil	BR17-90	DQ340412	CPV-2a	1990	Hap_44
19	Brazil	BR22-93	DQ340422	CPV-2a	1993	Hap_45
20	Brazil	BR46-95	DQ340430	CPV-2a	1995	Hap_46
21	Brazil	BR56-95	DQ340431	CPV-2a	1995	Hap_47
22	Brazil	BR7168-00	DQ340433	CPV-2a	2000	Hap_48
23	Brazil	BR8155-00	DQ340434	CPV-2a	2000	Hap_49
24	Brazil	Bel2014-01	KX774249	CPV-2b	2014	Hap_159
25	Brazil	Bel2016-01	KX774250	CPV-2b	2016	Hap_159
26	Brazil	Bel2015-01	KX774251	CPV-2b	2015	Hap_160
27	China	HN-3	DQ177497	CPV-2b	2005	Hap_39
28	China	RPPV	DQ354068	CPV-2a	UNK	Hap_50
29	China	B-2004	EF011664	CPV-2a	UNK	Hap_51
30	China	CPV/BJ004/07	EF666059	CPV-2a	UNK	Hap_55
31	China	CPV/BJ008/07	EF666061	CPV-2a	UNK	Hap_56
32	China	CPV/BJ17/06	EF666065	CPV-2a	UNK	Hap_57
33	China	CPV/BJ020/07	EF666067	CPV-2a	UNK	Hap_50
34	China	CPV/BJ050/07	EU145955	CPV-2a	UNK	Hap_63
35	China	CPV-SHZ	EU170352	CPV-2a	UNK	Hap_64
36	China	CPV-HZ0761	EU213073	CPV-2a	UNK	Hap_65
37	China	CPV-APD1	EU213074	CPV-2a	UNK	Hap_50
38	China	CPV-BD1 (2b)	EU213075	CPV-2b	UNK	Hap_66
39	China	CPV-JB1 (2a)	EU213076	CPV-2a	UNK	Hap_67
40	China	CPV-HT (2a)	EU213077	CPV-2a	UNK	Hap_68
41	China	CPV-BD3 (2a)	EU213080	CPV-2a	UNK	Hap_50
42	China	CPV-BD4 (2a)	EU213081	CPV-2a	UNK	Hap_69
43	China	CPV-KT1 (2a)	EU213082	CPV-2a	UNK	Hap_70
44	China	CPV-KT2 (2a)	EU213083	CPV-2a	UNK	Hap_70
45	China	CPV-ZD2 (2a)	EU213084	CPV-2a	UNK	Hap_71
46	China	CPV-ZD3 (2a)	EU213085	CPV-2a	UNK	Hap_72
47	China	cpv/nj01/06	EU310373	CPV-2a	2006	Hap_73
48	China	CPV/WH02/06	EU377537	CPV-2a	2006	Hap_74
49	China	PV/PL/HeN03/08	EU441279	CPV-2a	2008	Hap_75
50	China	PV/PL/HeN02/08	EU441280	CPV-2a	2008	Hap_76
51	China	CPV-JB3	EU483510	CPV-2b	UNK	Hap_77
52	China	CPV-ZD4	EU483511	CPV-2a	UNK	Hap_78
53	China	CPV-ZD6	EU483512	CPV-2b	UNK	Hap_10
54	China	CPV-ZD35	EU483517	CPV-2b	UNK	Hap_79
55	China	HLJ-JQ	EU697385	CPV-2a	2007	Hap_84
56	China	02/09	GU380299	CPV-2b	2009	Hap_112

57	China	04/09	GU380301	CPV-2a	2009	Hap_73
58	China	08/09	GU380305	CPV-2c	2009	Hap_113
59	China	09/09	GU452713	CPV-2a	2009	Hap_50
60	China	11/09	GU452715	CPV-2a	2009	Hap_114
61	China	JL0201	GU569946	CPV-2a	2001	Hap_115
62	China	CC8601	GU569948	CPV-2a	1986	Hap_50
63	China	YAZA1	HQ651237	CPV-2a	2009	Hap_128
64	China	BJ/2010	HQ883273	CPV-2a	2010	Hap_129
65	China	S2	JF767492	CPV-2a	2009	Hap_50
66	China	S5	JF767493	CPV-2a	2009	Hap_50
67	China	S7	JF767494	CPV-2a	2009	Hap_137
68	China	G12	JF795456	CPV-2a	2010	Hap_57
69	China	Shanaxi	JN403045	CPV-2a	2011	Hap_138
70	China	CPV-LZ2	JQ268284	CPV-2b	2011	Hap_145
71	China	CPV-YH	KY403998	CPV-2a	2008	Hap_25
72	China	CPV-BJ03/17	MF134808	CPV-2a	2017	Hap_170
73	China	Canine/China/08/2016	MF805796	CPV-2c	2016	Hap_171
74	China	CPV-BJL1	MH106698	CPV-2a	2015	Hap_25
75	China	CPV-BJL2	MH106699	CPV-2b	2015	Hap_145
76	China	CPV-BJL3	MH106700	CPV-2c	2016	Hap_172
77	China	Canine/China/13/2017	MH476580	CPV-2a	2017	Hap_173
78	China	Canine/China/12/2017	MH476581	CPV-2c	2017	Hap_30
79	China	Canine/China/11/2017	MH476582	CPV-2a	2017	Hap_174
80	China	Canine/China/15/2017	MH476584	CPV-2c	2017	Hap_175
81	China	Canine/China/16/2017	MH476585	CPV-2c	2017	Hap_171
82	China	Canine/China/17/2017	MH476586	CPV-2a	2017	Hap_25
83	China	Canine/China/19/2017	MH476588	CPV-2b	2017	Hap_145
84	China	Canine/China/20/2017	MH476589	CPV-2a	2017	Hap_176
85	China	Canine/China/21/2017	MH476590	CPV-2a	2017	Hap_25
86	China	Canine/China/23/2017	MH476592	CPV-2c	2017	Hap_177
87	China	Canine/China/24/2017	MH476593	CPV-2a	2017	Hap_178
88	China	CPV-AHhf1	MT010564	CPV-2c	2018	Hap_30
89	China	PV-2c/China/SC-14/2017	MT165693	CPV-2c	2017	Hap_171
90	Germany	CPV-447	AY742934	CPV-2b	1995	Hap_21
91	Germany	CPV-U6	AY742935	CPV-2a	1995	Hap_15
92	Germany	CPV-U51	AY742942	CPV-2c	1997	Hap_18
93	Germany	G7/97	FJ005196	CPV-2c	1997	Hap_85
94	Germany	FPV-377	U22188	FPV	1993	Hap_188
95	Greece	GR51/08	GQ865518	CPV-2c	2008	Hap_18
96	Greece	GR09/09	GQ865519	CPV-2c	2009	Hap_18
97	India	CPV2a	AJ564427	CPV-2a	UNK	Hap_19
98	India	TN/CPV2a/2018	MH545963	CPV-2a	2018	Hap_25
99	India	India CPV-2a	MN661243	CPV-2a	2016	Hap_186
100	Italy	CPV-W42	AF306444	CPV-2b	1995	Hap_13
101	Italy	CPV-632	AF306445	CPV-2a	1996	Hap_14
102	Italy	CPV-584	AF306446	CPV-2a	1994	Hap_15
103	Italy	CPV-618	AF306447	CPV-2a	1995	Hap_15
104	Italy	CPV-616	AF306449	CPV-2b	1995	Hap_13
105	Italy	CPV-637	AF306450	CPV-2b	1996	Hap_16
106	Italy	CPV-699	AF393506	CPV-2a	2000	Hap_17
107	Italy	CPV-695	AF401519	CPV-2c	UNK	Hap_18
108	Italy	219/08-5	FJ005249	CPV-2c	2008	Hap_86
109	Italy	219/08-13	FJ005250	CPV-2c	2008	Hap_87
110	Italy	239/08	FJ005251	CPV-2c	2008	Hap_88
111	Italy	331/05	FJ005254	CPV-2a	2005	Hap_89
112	Italy	,17/08	FJ005256	CPV-2a	2008	Hap_15
113	Italy	54/08	FJ005257	CPV-2a	2008	Hap_90
114	Italy	80/08	FJ005258	CPV-2a	2008	Hap_89

115	Italy	100/08	FJ005259	CPV-2a	2008	Hap_15
116	Italy	134/05	FJ005264	CPV-2b	2005	Hap_91
117	Italy	12/08-A	GU362933	CPV-2a	2008	Hap_111
118	Italy	12/08-B	GU362934	CPV-2a	2008	Hap_111
119	Italy	CPV-2c/707/2001	KF373575	CPV-2c	2001	Hap_18
120	Japan	CPV97-008	AB115504	CPV-2b	1997	Hap_8
121	Japan	Sho-nan	AB128923	CPV-2b	2003	Hap_12
122	Japan	Y1	D26079	CPV-2a	1982	Hap_37
123	Japan	FPV-314	D78585	CPV-2a	1993	Hap_38
124	Mongolia	5 MGL	MH660909	CPV-2c	2017	Hap_179
125	Nigeria	NIG001	HQ602990	CPV-2a	2010	Hap_123
126	Nigeria	NIG006	HQ602991	CPV-2a	2010	Hap_124
127	Nigeria	NIG002	HQ602992	CPV-2a	2010	Hap_125
128	Nigeria	NIG003	HQ602993	CPV-2a	2010	Hap_124
129	Nigeria	NIG004	HQ602994	CPV-2a	2010	Hap_126
130	Nigeria	15-10NGR2010	HQ602995	CPV-2a	2010	Hap_127
131	Nobivac®DHPPI	CPVint (vaccine)	FJ197846	CPV-2	N/A	Hap_109
132	NZ	CPV-339	AY742933	CPV-2a	1993	Hap_20
133	NZ	CPV026	KP881620	CPV-2a	2010	Hap_148
134	NZ	CPV082	KP881621	CPV-2a	2010	Hap_149
135	NZ	CPV040	KP881626	CPV-2a	2010	Hap_150
136	NZ	CPV081	KP881631	CPV-2a	2010	Hap_151
137	NZ	CPV084	KP881633	CPV-2a	2010	Hap_149
138	NZ	CPV031	KP881638	CPV-2a	2010	Hap_149
139	NZ	CPV036	KP881639	CPV-2a	2010	Hap_152
140	NZ	CPV068	KP881641	CPV-2a	2010	Hap_149
141	NZ	MAF3	KP881645	CPV-2	1986	Hap_80
142	NZ	CPV030	KP881646	CPV-2a	2010	Hap_149
143	NZ	CPV075	KP881647	CPV-2a	2010	Hap_153
144	NZ	MAF1	KP881649	CPV-2a	2009	Hap_154
145	NZ	CPV006	KP881652	CPV-2a	2009	Hap_149
146	NZ	CPV039	KP881654	CPV-2a	2010	Hap_151
147	NZ	CPV080	KP881656	CPV-2a	2010	Hap_151
148	NZ	CPV079	KP881662	CPV-2a	2010	Hap_155
149	NZ	CPV038	KP881664	CPV-2a	2010	Hap_151
150	NZ	CPV003	KP881667	CPV-2a	2009	Hap_149
151	NZ	CPV009	KP881669	CPV-2a	2009	Hap_156
152	NZ	CPV071	KP881670	CPV-2a	2010	Hap_157
153	NZ	CPV001	KP881674	CPV-2a	2009	Hap_151
154	NZ	CPV019	KP881677	CPV-2a	2010	Hap_157
155	NZ	CPV022	KP881678	CPV-2a	2010	Hap_153
156	NZ	CPV041	KP881680	CPV-2a	2010	Hap_158
157	NZ	MAF2	KP881684	CPV-2a	1990	Hap_40
158	Poland	pVP246	Z46651	CPV-2b	UNK	Hap_4
159	South Africa	22-10SA 2010	HQ602969	CPV-2b	2010	Hap_4
160	South Africa	68-10SA 2010	HQ602970	CPV-2b	2010	Hap_116
161	South Africa	3-10SA 2010	HQ602971	CPV-2a	2010	Hap_117
162	South Africa	32-10SA 2010	HQ602972	CPV-2b	2010	Hap_118
163	South Africa	108-10SA 2010	HQ602973	CPV-2a	2010	Hap_119
164	South Africa	31-10SA 2010	HQ602974	CPV-2a	2010	Hap_117
165	South Africa	101-10SA 2010	HQ602975	CPV-2a	2010	Hap_50
166	South Africa	72-10SA 2010	HQ602976	CPV-2b	2010	Hap_116
167	South Africa	100-10SA 2010	HQ602977	CPV-2a	2010	Hap_50
168	South Africa	63-10SA 2010	HQ602978	CPV-2b	2010	Hap_120
169	South Africa	67-10SA 2010	HQ602979	CPV-2b	2010	Hap_116
170	South Africa	33-10SA 2010	HQ602980	CPV-2b	2010	Hap_116
171	South Africa	78-10SA 2010	HQ602981	CPV-2b	2010	Hap_116
172	South Africa	85-10SA 2010	HQ602982	CPV-2b	2010	Hap_116

173	South Africa	86-10SA 2010	HQ602983	CPV-2b	2010	Hap_116
174	South Africa	89-10SA 2010	HQ602984	CPV-2b	2010	Hap_116
175	South Africa	8-10SA 2010	HQ602985	CPV-2b	2010	Hap_116
176	South Africa	98-10SA 2010	HQ602986	CPV-2b	2010	Hap_121
177	South Africa	99-10SA 2010	HQ602987	CPV-2b	2010	Hap_122
178	South Africa	CPV-2 2010	HQ602989	CPV-2	2010	Hap_110
179	South Korea	CPVKK0501	EF189717	CPV-2a	UNK	Hap_52
180	South Korea	DH326	EF599097	CPV-2b	2005	Hap_53
181	South Korea	Pome	EF599098	CPV-2a	2005	Hap_54
182	South Korea	K001	EU009200	CPV-2a	2003 – 2006	Hap_58
183	South Korea	K014	EU009201	CPV-2a	2003 – 2006	Hap_59
184	South Korea	K015	EU009202	CPV-2a	2003 – 2006	Hap_60
185	South Korea	K022	EU009203	CPV-2a	2003 – 2006	Hap_61
186	South Korea	K026	EU009204	CPV-2a	2003 – 2006	Hap_50
187	South Korea	K029	EU009205	CPV-2b	2003 – 2006	Hap_4
188	South Korea	K031	EU009206	CPV-2b	2003 – 2006	Hap_62
189	South Korea	CPVK1	FJ197823	CPV-2a	2007	Hap_92
190	South Korea	CPVK2	FJ197824	CPV-2a	2007	Hap_93
191	South Korea	CPVK3	FJ197825	CPV-2a	2007	Hap_94
192	South Korea	CPVK4	FJ197826	CPV-2a	2007	Hap_60
193	South Korea	CPVK5	FJ197827	CPV-2a	2007	Hap_95
194	South Korea	CPVK6	FJ197828	CPV-2a	2007	Hap_96
195	South Korea	CPVK7	FJ197829	CPV-2a	2007	Hap_97
196	South Korea	CPVK8	FJ197830	CPV-2a	2007	Hap_60
197	South Korea	CPVK9	FJ197831	CPV-2a	2007	Hap_98
198	South Korea	CPVK10	FJ197832	CPV-2a	2007	Hap_58
199	South Korea	CPVK11	FJ197833	CPV-2a	2007	Hap_99
200	South Korea	CPVK12	FJ197834	CPV-2a	2007	Hap_89
201	South Korea	CPVK13	FJ197835	CPV-2a	2007	Hap_100
202	South Korea	CPVK14	FJ197836	CPV-2a	2007	Hap_101
203	South Korea	CPVK15	FJ197837	CPV-2a	2007	Hap_58
204	South Korea	CPVK16	FJ197838	CPV-2a	2007	Hap_102
205	South Korea	CPVK17	FJ197839	CPV-2a	2007	Hap_103
206	South Korea	CPVK18	FJ197840	CPV-2a	2007	Hap_104
207	South Korea	CPVK19	FJ197841	CPV-2a	2007	Hap_105
208	South Korea	CPVK20	FJ197842	CPV-2a	2007	Hap_106
209	South Korea	CPVK21	FJ197843	CPV-2a	2007	Hap_107
210	South Korea	CPVK22	FJ197844	CPV-2a	2007	Hap_60
211	South Korea	CPVK23	FJ197845	CPV-2a	2007	Hap_108
212	South Korea	Korea CPV2c_1	MK306289	CPV-2c	2017	Hap_180
213	South Korea	Korea CPV2c_2	MK306290	CPV-2c	2017	Hap_181
214	South Korea	Gigucheon	MN400978	FPV	2017	Hap_182
215	South Korea	Jun	MN400979	FPV	2017	Hap_183
216	South Korea	Rara	MN400980	FPV	2017	Hap_184
217	South Korea	Rachi	MN400981	CPV-2b	2017	Hap_185
218	Sri Lanka	CPV.S02	CPV/S02	CPV-2a	2015-17	Hap_25
219	Sri Lanka	CPV.S03	CPV/S03	CPV-2b	2015-17	Hap_26
220	Sri Lanka	CPV.S05	CPV/S05	CPV-2a	2015-17	Hap_25
221	Sri Lanka	CPV.S15	CPV/S15	CPV-2a	2015-17	Hap_27
222	Sri Lanka	CPV.S16	CPV/S16	CPV-2b	2015-17	Hap_26
223	Sri Lanka	CPV.S18	CPV/S18	CPV-2b	2015-17	Hap_28
224	Sri Lanka	CPV.S19	CPV/S19	CPV-2a	2015-17	Hap_25
225	Sri Lanka	CPV.S25	CPV/S25	CPV-2c	2015-17	Hap_29
226	Sri Lanka	CPV.S26	CPV/S26	CPV-2c	2015-17	Hap_29
227	Sri Lanka	CPV.S28	CPV/S28	CPV-2c	2015-17	Hap_30
228	Sri Lanka	CPV.S29	CPV/S29	CPV-2c	2015-17	Hap_30
229	Sri Lanka	CPV.S31	CPV/S31	CPV-2c	2015-17	Hap_30
230	Sri Lanka	CPV.S34	CPV/S34	CPV-2a	2015-17	Hap_31

231	Sri Lanka	CPV.S39	CPV/S39	CPV-2a	2015-17	Hap_31
232	Sri Lanka	CPV.S40	CPV/S40	CPV-2a	2015-17	Hap_31
233	Sri Lanka	CPV.S41	CPV/S41	CPV-2a	2015-17	Hap_31
234	Sri Lanka	CPV.S42	CPV/S42	CPV-2a	2015-17	Hap_32
235	Sri Lanka	CPV.S43	CPV/S43	CPV-2c	2015-17	Hap_30
236	Sri Lanka	CPV.S45	CPV/S45	CPV-2a	2015-17	Hap_25
237	Sri Lanka	CPV.S46	CPV/S46	CPV-2b	2015-17	Hap_33
238	Sri Lanka	CPV.S47	CPV/S47	CPV-2	2015-17	Hap_34
239	Sri Lanka	CPV.S48	CPV/S48	CPV-2b	2015-17	Hap_33
240	Sri Lanka	CPV.S49	CPV/S49	CPV-2a	2015-17	Hap_25
241	Sri Lanka	CPV.S50	CPV/S50	CPV-2a	2015-17	Hap_25
242	Sri Lanka	CPV.S52	CPV/S52	CPV-2a	2015-17	Hap_35
243	Sri Lanka	CPV.S54	CPV/S54	CPV-2a	2015-17	Hap_25
244	Sri Lanka	CPV.S55	CPV/S55	CPV-2a	2015-17	Hap_36
245	Sri Lanka	CPV.S56	CPV/S56	CPV-2c	2015-17	Hap_30
246	Sri Lanka	CPV.S57	CPV/S57	CPV-2a	2015-17	Hap_36
247	Sri Lanka	CPV.S58	CPV/S58	CPV-2c	2015-17	Hap_30
248	Sri Lanka	CPV.S61	CPV/S61	CPV-2a	2015-17	Hap_36
249	Taiwan	Taiwan9	AB054213	CPV-2a	1998	Hap_1
250	Taiwan	Taichung	AY869724	CPV-2b	2004	Hap_24
251	Taiwan	T10 (2b)	U72696	CPV-2b	1995	Hap_8
252	Thailand	CPV/VT114	KP715709	CPV-2b	UNK	Hap_147
253	UK	FPV-a	M24002	FPV	1962	Hap_166
254	Uruguay	Uy-243/10	JF906788	CPV-2a	2010	Hap_25
255	Uruguay	M21	KC196099	CPV-2c	2006	Hap_146
256	USA	CPV-193	AY742932	CPV-2b	1991	Hap_4
257	USA	CPV-431	AY742951	CPV-2b	2003	Hap_22
258	USA	CPV-435	AY742953	CPV-2a	2003	Hap_23
259	USA	CPV-5.us.79	EU659116	CPV-2	1979	Hap_80
260	USA	CPV-6.us.80	EU659117	CPV-2	1980	Hap_80
261	USA	CPV-13.us.81	EU659118	CPV-2b	1981	Hap_81
262	USA	CPV-410.us.00	EU659119	CPV-2b	2000	Hap_82
263	USA	CPV-411a.us.98	EU659120	CPV-2b	1998	Hap_82
264	USA	USA010	EU659121	CPV-2b	1998	Hap_83
265	USA	CPV-2b/Dog/CA/148743/08	JN867602	CPV-2b	2008	Hap_139
266	USA	CPV-2b/Dog/KS/81213/09	JN867603	CPV-2b	2009	Hap_140
267	USA	CPV-2b/Dog/US/142805/09	JN867605	CPV-2b	2009	Hap_142
268	USA	CPV-2b/Dog/US/19923/09	JN867606	CPV-2b	2009	Hap_53
269	USA	CPV-2b/Dog/NY/LP62/08	JN867607	CPV-2b	2008	Hap_143
270	USA	CPV-2b/Dog/IL/24124/09	JN867608	CPV-2b	2009	Hap_141
271	USA	CPV-2b/Dog/KY/93238/08	JN867609	CPV-2b	2008	Hap_144
272	USA	FPV-Carlson	M10824	FPV	1966	Hap_163
273	USA	CPV_Norden	M19296	CPV-2	1978	Hap_164
274	USA	CPV-31	M24000	CPV-2a	1983	Hap_165
275	USA	CPV-15	M24003	CPV-2a	1984	Hap_167
276	USA	CPV-b	M38245	CPV-2	1978	Hap_80
277	USA	FPV-b	M38246	FPV	1967	Hap_168
278	USA	CPV-39	M74849	CPV-2b	1984	Hap_169
279	USA	CPV-133	M74852	CPV-2b	1990	Hap_4
280	USA	CPV39	MN451663	CPV-2b	1984	Hap_4
281	USA	CPV-128	U22186	CPV-2	1979	Hap_80
282	USA	FPV-23	U22187	FPV	1990	Hap_187
283	USA	FPV-d	U22189	FPV	1964	Hap_189
284	USA	CPV-2b/Dog/IL/137654/08	JN867604	CPV-2b	2008	Hap_141
285	Vanguard®plus5	CPVpf (vaccine)	FJ197847	CPV-2	N/A	Hap_110
286	Vietnam	V120	AB054215	CPV-2a	1997	Hap_2
287	Vietnam	V154	AB054217	CPV-2a	1997	Hap_3
288	Vietnam	V209	AB054219	CPV-2b	1997	Hap_4

289	Vietnam	V217	AB054220	CPV-2b	1997	Hap_4
290	Vietnam	LCPVV204	AB054221	CPV-2b	1997	Hap_5
291	Vietnam	LCPVV139	AB054222	CPV-2a	1997	Hap_6
292	Vietnam	LCPVV203	AB054224	CPV-2b	1997	Hap_7
293	Vietnam	HCM-8	AB120721	CPV-2b	2003	Hap_9
294	Vietnam	HNI-2-13	AB120724	CPV-2b	2003	Hap_10
295	Vietnam	HNI-4-1	AB120727	CPV-2c	2003	Hap_11
296	Vietnam	CPV/dog/HCM/14/2013	LC216909	CPV-2c	2013	Hap_161
297	Vietnam	CPV/dog/HCM/20/2013	LC216910	CPV-2c	2013	Hap_162
